# Prevalence of menstrual pain and symptoms and their association with age and BMI among Japanese female app users

**DOI:** 10.1038/s41598-025-02459-0

**Published:** 2025-05-28

**Authors:** Shuxian Liu, Daisuke Yoneoka, Hitomi Suzuki, Kiriko Sasayama, Eiko Saito, Yuna Naraoka, Momo Hosokawa, Ataru Igarashi, Erika Ota

**Affiliations:** 1 Global Health Nursing, Graduate School of Nursing Science, St. Luke’s International University, Tokyo, Japan; 2 Center for Surveillance, Immunization, and Epidemiologic Research, National Institute of Infectious Diseases, Tokyo, Japan; 3 International Healthcare and Midwifery, Graduate School of Nursing, Japanese Red Cross College of Nursing, Tokyo, Japan; 4 Sustainable Society Design Center, Graduate School of Frontier Sciences, The University of Tokyo, Chiba, Japan; 5 Graduate School of Medicine, Intractable Disease Research Center, Juntendo University, Tokyo, Japan; 6 Japanese Center for Research on Woman in Sport, Juntendo University, Tokyo, Japan; 7 General Incorporated Association Luvtelli, Tokyo, Japan; 8 Department of Health Economics and Outcomes Research, Graduate School of Pharmaceutical Sciences, The University of Tokyo, Tokyo, Japan

**Keywords:** Public health, Health care, Risk factors

## Abstract

Menstruation plays a crucial role in women’s health, with age and BMI potentially influencing menstrual pain and associated symptoms. This cross-sectional study analyzed data from Japanese female Sofy app users aged 12–51 years, collected between March 4, 2021, and August 17, 2023. After excluding duplicate and incomplete responses, as well as participants using contraceptives or painkillers, a total of 32,556 valid responses were included. Among the participants, 66.83% reported experiencing menstrual pain, with 28.33% describing it as mild, 25.13% as moderate, and 13.31% as severe. The average number of symptoms reported was 3.36, including 2.43 physical and 0.82 emotional symptoms. Abdominal pain and depression were the most common physical and emotional symptoms, respectively. Menstrual pain and the number of symptoms exhibited an inverted U-shaped relationship with age, peaking slightly earlier in life, and a U-shaped relationship with BMI. Participants in the normal BMI range reported the lowest prevalence of menstrual pain, while those who were underweight had the highest prevalence. Generalized Additive Models (GAM) revealed significant interactions between age and BMI for both menstrual pain and the number of symptoms (p < 0.0001). These findings revealed the age-related inverted U-shape and BMI-related U-shape patterns across all outcomes. Menstrual pain prevalence peaked around the mid-twenties, and symptoms decreased as BMI approached the normal range. The findings provide valuable insights into identifying high-risk groups and informing the development of targeted interventions to improve women’s health.

## Introduction

Menstruation is an integral and essential aspect of women’s overall health^1^. From a life course perspective, it encompasses physical, psychological, and social health issues spanning from pre-menarche to post-menopause, necessitating further recognition, framing, and addressing^2^. During menstruation, many women experience a variety of physical symptoms such as pain, as well as emotional symptoms like anxiety and depression, which affect their quality of life and social well-being^1,3^.

The most common discomfort experienced during menstruation is menstrual pain, also known as dysmenorrhea, which is defined as painful menstrual cramps originating from the uterus. It is also the most common gynecological disorder among women of reproductive age^4^. In addition to menstrual pain, women during menstruation often experience accompanying symptoms such as nausea and vomiting, fatigue, headaches, dizziness, and other symptoms ^5^, which can affect mental health and lead to emotional symptoms like anxiety and depression ^3^. Moreover, due to hormonal changes and increased pain sensitivity, women may be more susceptible to headache and other pain related symptoms during menstruation^6,7^. These physical and emotional symptoms not only increase the suffering of menstruating women but also impact their regular daily activities^8^, quality of life^9^, and sleep quality^10^. Additionally, menstruation-related symptoms can affect women’s concentration, reduce academic and work performance, and affect attendance, leading to a range of social and economic impacts^11^.

There are many potential influencing factors for menstrual pain and menstruation-related symptoms. Obesity may be related to menstrual disorders by affecting endometrial repair and hormonal changes, increasing the incidence of menstrual irregularities and polycystic ovary syndrome^12^. Although study results vary, existing research indicates that being underweight, overweight, or obese may be potentially associated with dysmenorrhea and other menstruation-related symptoms^13^. Additionally, current studies have indicated the relationship between age and the occurrence and distribution of menstrual pain and menstruation-related symptoms^14^.

Current studies have reported the prevalence of menstrual pain and menstrual symptoms, as well as their associations with age and BMI. However, existing research presents some limitations: (1) The majority of studies have a sample size of less than 20,000, and there are very few studies conducted on large sample size. A study based on the Flo app reported results based on 19,266,573 users; however, its primary aim was to document menstrual patterns in women and only reported differences in the prevalence and number of menstrual symptoms across different age groups^15^. (2) Many studies focus solely on menstrual pain, neglecting other menstrual symptoms that can similarly have adverse effects on women’s health; (3) When investigating the relationship between age, BMI, and menstrual pain and symptoms, most studies simply compare differences across age and BMI groups^15–17^, overlooking the potential nonlinear relationships between age and BMI.

Additionally, the majority of current research is centered on the countries in the Americas^16,18^, Europe^19^, Australia^20^ and West Asia^21^, with relatively few studies focusing only on Japanese women^13,22^. Therefore, conducting a large-scale cross-sectional study to comprehensively understand the prevalence of menstrual pain and related symptoms among Japanese women, while examining their distribution across different age and BMI groups and exploring the potential nonlinear relationships with age and BMI, is crucial. Such an analysis is essential for advancing the understanding of menstrual pain and related symptoms, particularly within the Japanese female population.

The purposes of this study include (1) To investigate the prevalence of menstrual pain and other menstrual symptoms in the Japanese female app users; (2) To clarify the basic characteristics of menstruation-related symptoms, including physical and emotional symptoms, across different age groups and BMI categories; (3) To clarify the basic characteristics of the total number of menstruation symptoms, menstruation physical symptoms, and menstruation emotion symptoms among Japanese women, as well as their distribution across different age groups and BMI categories; and (4) To estimate the non-linear association between BMI and age with menstrual pain, the total number of menstruation-related symptoms, the number of menstruation-related physical symptoms, and the number of menstruation-related emotion symptoms.

## Method

### Data collection

This cross-sectional study, conducted through the Sofy App (Unicharm Corporation Co., Ltd, Tokyo, Japan), and include all records entered into the apps from 4th March, 2021 up to 17th August 2023. Sofy is a well-known and leading brand in the sanitary product sector in Japan, with a large consumer base. The Sofy app provides tools for women to manage and record menstrual data and symptoms, offering advice and support for menstrual discomfort. Currently, these apps have over one million users in Japan^23^.

The menstrual pain, menstrual symptoms, and additional information, including height, weight, BMI (calculated by dividing an individual’s weight in kilograms by the square of their height in meters), age, contraceptive use, and painkiller use, were collected through a self-reported mandatory sign-up questionnaire.

Menstrual pain was assessed using a self-reported four-point Likert scale, with severity levels ranging from 1 to 4, corresponding to no pain, mild pain, moderate pain, and severe pain, respectively. Other menstruation-related symptoms were classified into physical and emotion categories. Participants indicated the presence of these symptoms by selecting them on a checklist. The physical symptoms included 13 items: short-term and long-term fatigue, stomach ache, rough skin, sleepiness, breast distending pain, headache, lower back pain, chills, edema, increased appetite, nausea, and dizziness. The emotional symptoms included irritation, easily angered feelings, depression, anxiety, and emotion fluctuations. Additionally, the number of all symptoms, the number of physical symptoms, and the number of emotional symptoms were calculated to facilitate data analysis (with total counts of 18, 13, and 5, respectively). The use of contraceptives and painkillers was assessed through two questions: “Do you take contraceptives?” and “Do you take painkillers?” Participants who answered “yes” were considered to be using contraceptives or painkillers and were excluded from the analysis.

**Fig. 1:**
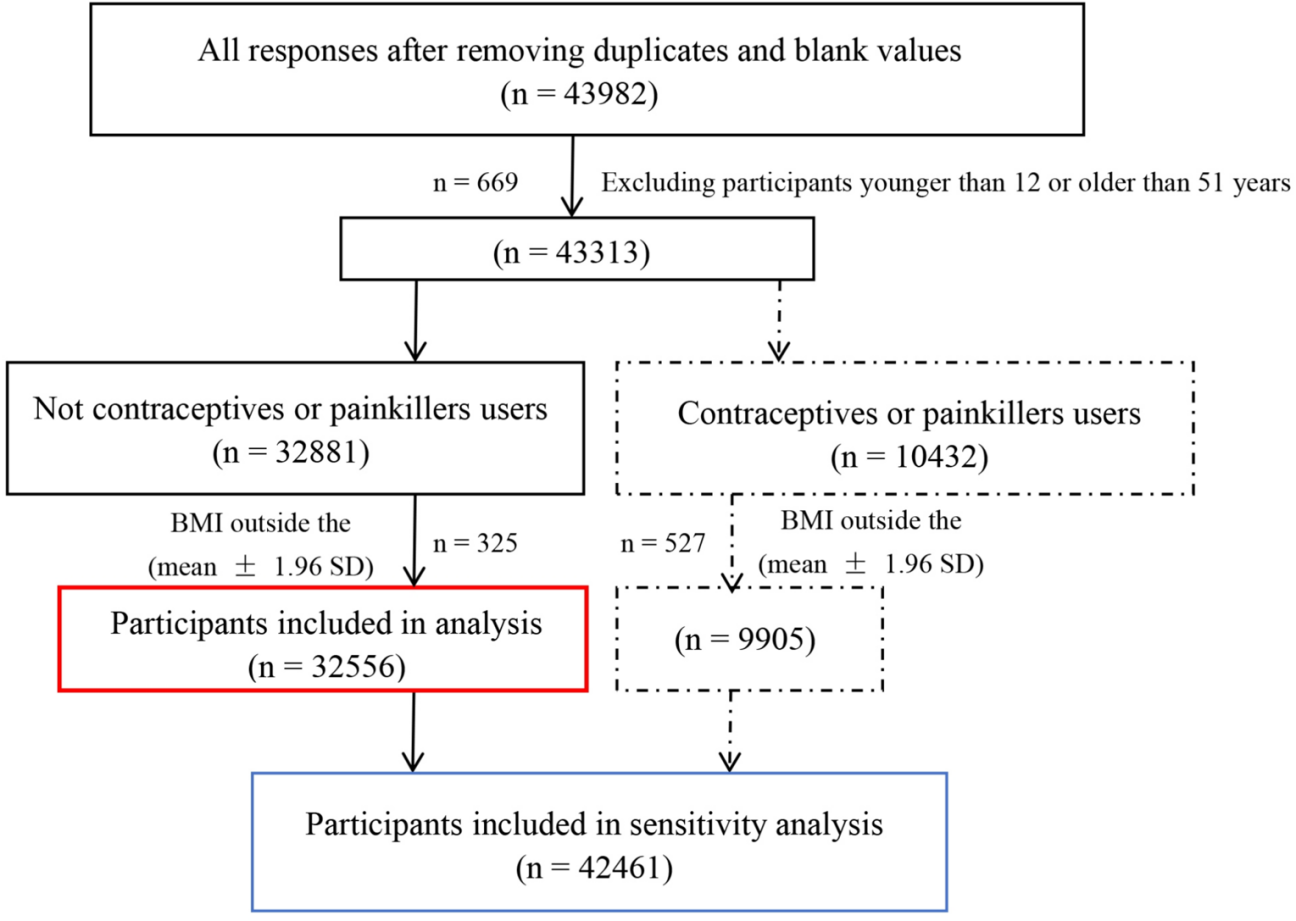
Inclusion and exclusion process for all responses.

All responses were imported into Excel, and those that did not meet the inclusion and exclusion criteria were subsequently removed. The inclusion and exclusion criteria are as follows. The inclusion criteria were (1) Japanese speaking; (2) Resides in Japan; and (3) Aged between 12 and 51 years old. The included age range is based on the median ages of menarche and menopause in Japan, which are 12.19^24^ and 50.54^25^, respectively. The exclusion criteria were (1) Participants taking contraceptive pills or painkillers, due to the potential relationship between these medication and menstrual symptoms^26,27^; (3) Duplicate user IDs; (4) Incomplete responses; and (5) Responses with BMI values outside the overall mean ± 1.96 standard deviation (SD). The inclusion and exclusion process for all responses was detailed in Fig. 1.

Ethical approval for this study protocol was obtained from the Shiba Palace Clinic Ethics Review Committee (approval number 153464_rn-36145). All methods were performed in accordance with the relevant guidelines and regulations. Informed consent was obtained from all individual participants included in the study.

### Statistical methods

The basic characteristics of BMI categories, severity of menstrual pain, and the number of symptoms for the overall population and each age group were summarized in the table in the form of N (%) and mean (SD). The prevalence of menstrual pain and the mean values of menstrual symptoms (including the number of all symptoms, physical symptoms, and emotion symptoms) across the age groups and BMI categories, along with their 95% confidence intervals (CIs), were calculated and visualized to observe their overall trends. The prevalence of different symptoms in the total population was calculated, and the prevalence distribution across different age groups and BMI categories were visualized using radar charts.

Generalized Additive Models (GAMs) were used to explore potential nonlinear and interactive relationships between age, BMI, and four symptom-related outcomes: menstrual pain, total symptoms, physical symptoms, and emotional symptoms. Unlike traditional regression models, GAMs can flexibly capture complex patterns in data without assuming a specific shape of the relationship. In this study, both age and BMI were included as continuous variables, and their interaction was modeled using smooth curves based on cubic splines. Menstrual pain, a binary outcome, was analyzed using logistic GAMs, while the three count-based outcomes were modeled using quasi-Poisson distributions to address overdispersion. Optimization for the model was conducted using restricted maximum likelihood (REML) approach. To guarantee the goodness of fitting, we compared its performance with other models (e.g., linear and spline-based regressions) based on AIC and BIC. GAM provided the best overall model fit based on AIC, with only minor differences in BIC. Full details of model comparisons are provided in Supplementary Material 1.

To evaluate the robustness of the findings, we also conducted a sensitivity analysis including participants who had been excluded due to the use of hormonal contraceptives or painkillers. Baseline characteristics were compared between groups, and the primary GAM models were re-estimated using the expanded sample to assess consistency of results.

All analyses were conducted using R version 4.3.3 with the following R packages: *fmsb* for the radar charts and *mgcv* for GAM.

## Results

After removing duplicates and incomplete submissions, a total of 32,556 valid responses were extracted from the apps for the analysis. The most represented age group was 32–41 years, comprising 9,445 participants, representing 29.0% of the sample. The majority of participants, 22,746 (69.87%), were classified as having a normal BMI. The overall prevalence of menstrual pain was 66.83%, with 28.33% reporting mild pain, 25.13% moderate pain, and 13.31% severe pain.

Participants reported an average of 3.36 menstruation-related symptoms, with a SD of 2.66, including an average of 2.43 physical symptoms (SD: 1.92) and 0.82 emotional symptoms (SD: 1.07). The age group of 22–31 years reported the highest prevalence of menstrual pain and the greatest number of menstruation-related, physical, and emotional symptoms. In contrast, participants aged 42–51 years reported the lowest levels in these categories. The basic demographic characteristics of the participants and a summary of the data were presented in Table 1.

**Table 1:** Characteristics of participants and summary of the data.

	All ages (N = 32,556)	12–21 years (N = 6724)	22–31 years (N = 8007)	32–41 years (N = 9445)	42–51 years (N = 8380)
BMI, n (row%)					
< 18.5	5641 (17.33%)	176 (26.29%)	1611 (20.12%)	1409 (14.92%)	853 (10.18%)
18.5–24.9	22,746 (69.87%)	4591 (68.28%)	5614 (70.11%)	6619 (70.08%)	5922 (70.67%)
25–29.9	4083 (12.54%)	360 (5.35%)	766 (9.57%)	1386 (14.67%)	1571 (18.75%)
> 30	86 (0.26%)	5 (0.07%)	16 (0.20%)	31 (0.33%)	34 (0.41%)
Menstrual pain, n (row%)					
No menstrual pain	8094 (24.86%)	1780 (26.47%)	1561 (19.50%)	2271 (24.04%)	2482 (29.62%)
Mild menstrual pain	9224 (28.33%)	1840 (27.36%)	2198 (27.45%)	2750 (29.12%)	2436 (29.07%)
Moderate menstrual pain	8181 (25.13%)	1755 (26.10%)	2313 (28.89%)	2399 (25.40%)	1714 (20.45%)
Severe menstrual pain	4352 (13.37%)	1134 (16.86%)	1405 (17.55%)	1129 (11.95%)	684 (8.16%)
Unknown	2705 (8.31%)	215 (3.20%)	530 (6.62%)	896 (9.49%)	1064 (12.70%)
Numbers of symptoms, mean (SD)					
Number of all symptoms	3.36 (2.66)	3.35 (2.87)	3.74 (2.66)	3.45 (2.61)	2.86 (2.42)
Number of physical symptoms	2.43 (1.92)	2.45 (2.07)	2.73 (1.96)	2.46 (1.89)	2.09 (1.74)
Number of emotional symptoms	0.82(1.07)	0.86 (1.14)	0.89 (1.08)	0.85 (1.07)	0.7 (0.97)

**Fig. 2:**
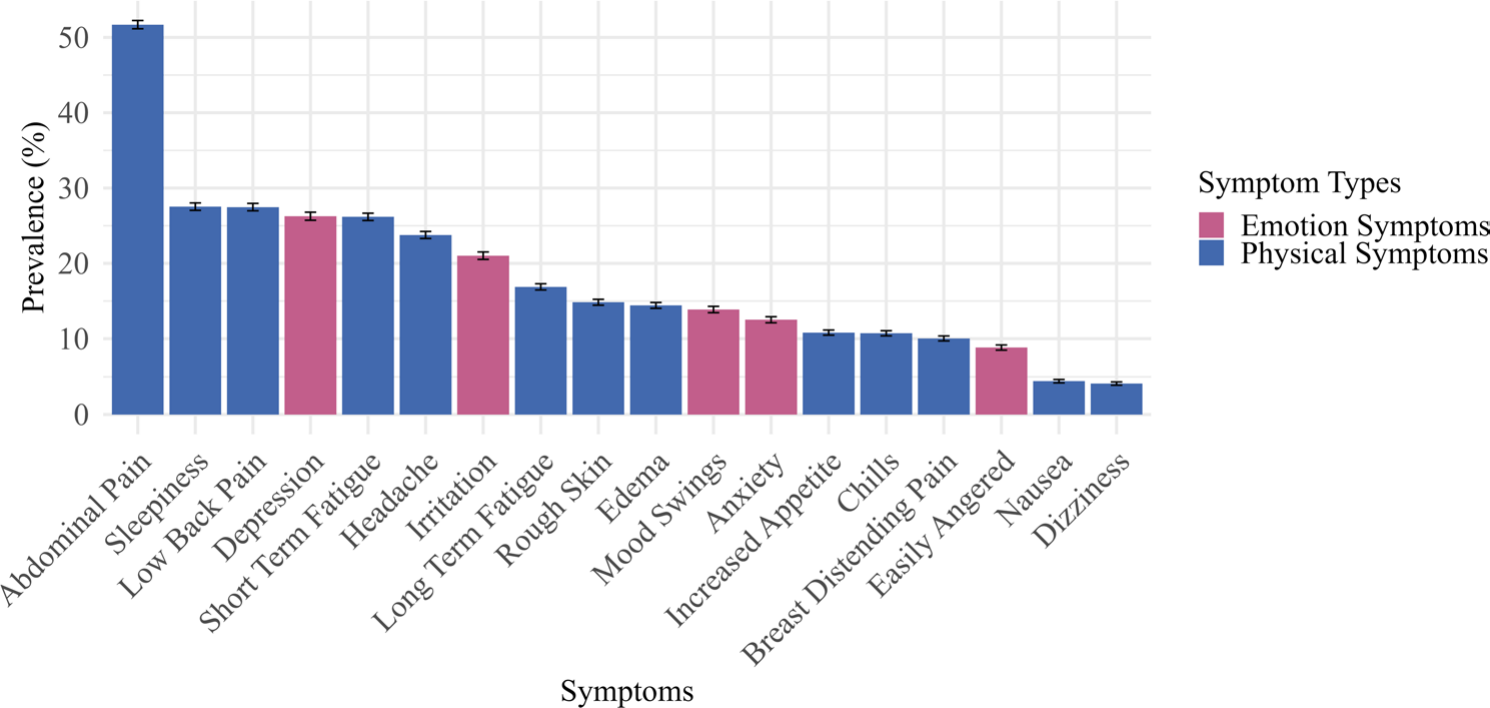
Bar chart of the prevalence of menstrual physical and emotional symptoms with 95% CIs.

The prevalence distribution of menstrual symptoms in the overall population was shown in Fig. 2. Among these symptoms, abdominal pain was the most common, followed by fatigue and lower back pain. Depression was the most frequently reported emotional symptom, with dizziness and nausea being the least common menstrual symptoms.

1. Distribution of menstrual pain prevalence and symptoms by age and BMI.

**Fig. 3:**
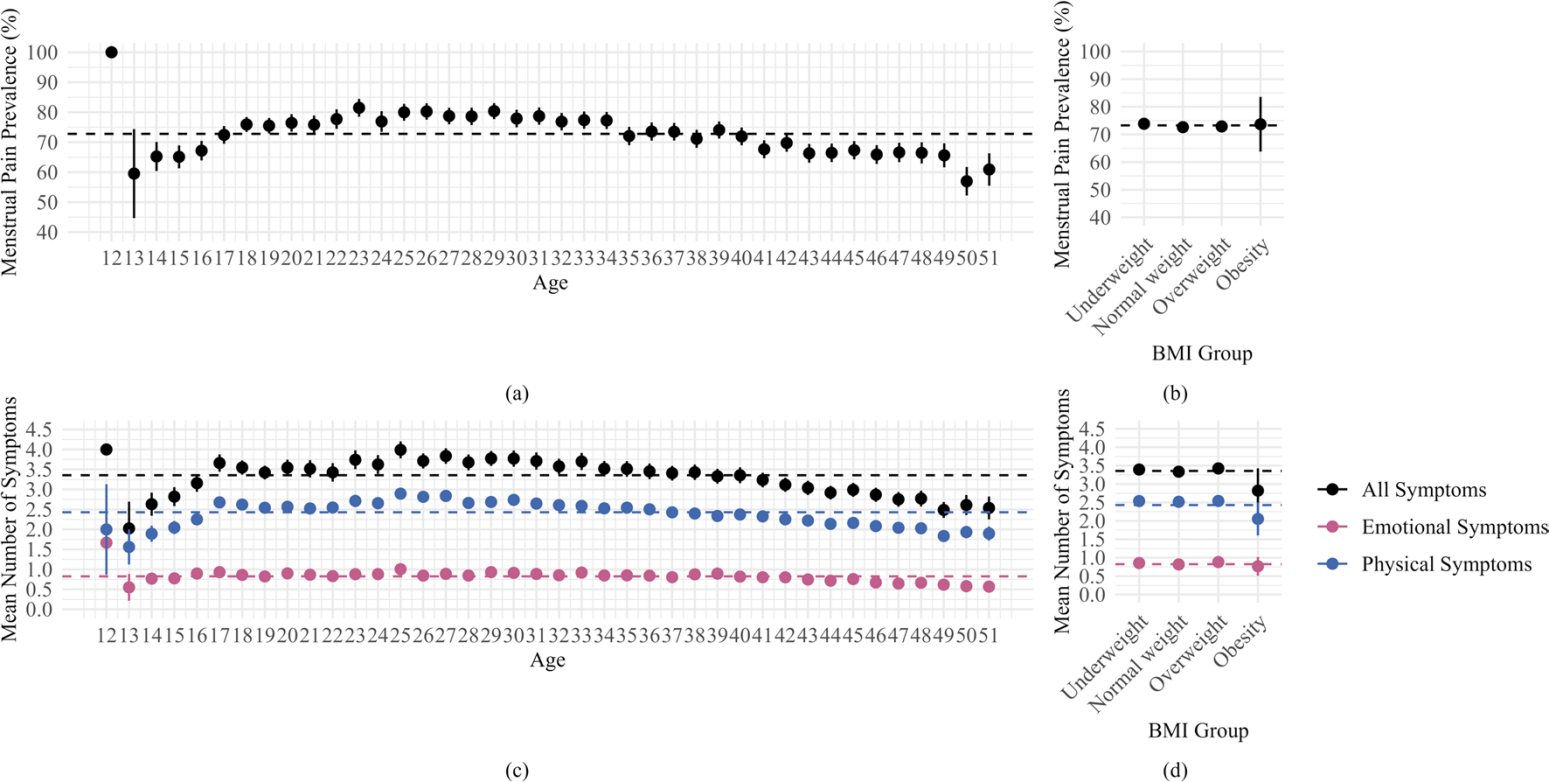
The distribution of menstrual pain across different age groups (**a**) and BMI categories (**b**), and the number of menstrual symptoms across different age groups (**c**) and BMI categories (**d**) with 95% CI.

Figure 3 illustrated the distribution of menstrual pain across different age groups (a) and BMI categories (b), and the number of menstrual symptoms across different age groups (c) and BMI categories (d). The prevalence of menstrual pain increased from age 12, peaks at 23 with over 81.47%, and then gradually declines. Menstrual pain was least common in individuals with normal weight, higher in those overweight, and most prevalent in those underweight. The number of menstrual symptoms increased with age, peaking at age 25. Moreover, the average number of symptoms was lowest in the obesity group, followed by the normal weight group. Additionally, the relatively wide 95% confidence interval observed in the obese group might be attributed to the very small proportion of obese individuals in the sample (0.26%).

**Fig. 4:**
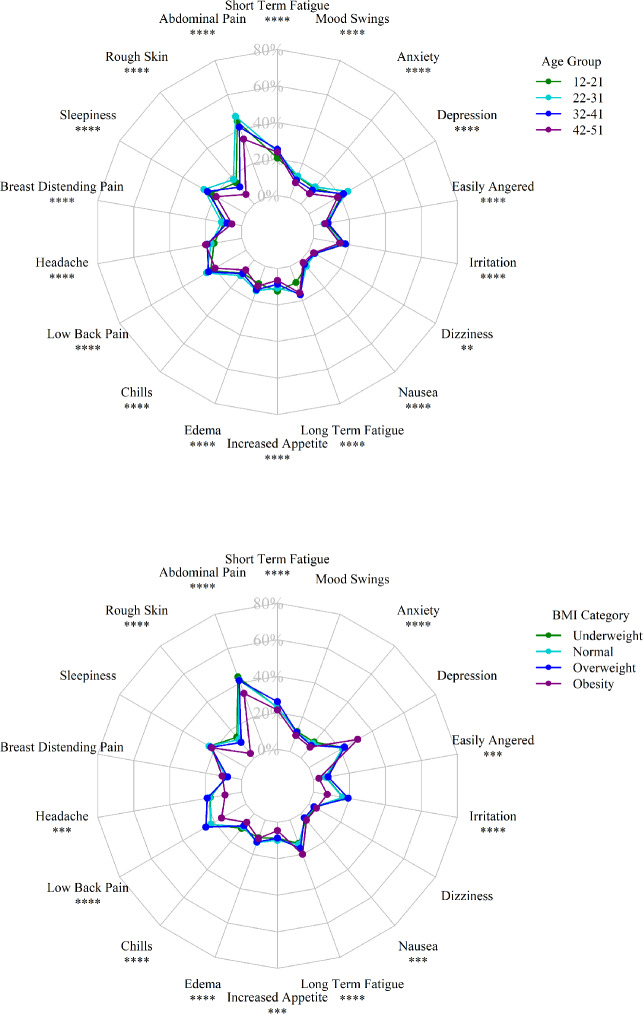
Symptom prevalence distribution radar chart: (**a**) radar chart showing symptom prevalence distribution across age groups; (**b**) radar chart showing symptom prevalence distribution across BMI groups. (The values for the 95% CI of the prevalence were provided in the Supplementary material 2 and p-values of various symptoms across different age and BMI groups were provided in the Supplementary material 3. Note: * indicates p < 0.05; ** p < 0.01; *** p < 0.001; **** p < 0.0001 (Chi-square test across age or BMI groups).

Radar charts (Fig. 4) illustrated the distribution of each menstrual symptom by age group and BMI category. In most symptoms, the 22–31 age group exhibited the highest prevalence, while the 42–51 age group had the lowest, as seen with symptoms like abdominal pain, rough skin, sleepiness, breast distending pain, chills, edema, nausea, dizziness, depression, anxiety, and mood swings. However, the prevalence of headaches was highest in the 42–51 age group, with progressively lower rates observed in younger groups. Compared to physical symptoms, emotional symptoms exhibited less variation across age groups.

In terms of BMI categories, obese individuals generally exhibited the lowest prevalence for most symptoms, except for depression (38.46%), breast distending pain (13.46%), long-term fatigue (25.00%), nausea (5.77%), and dizziness (5.77%), where they showed the highest rates. Overweight individuals had the highest prevalence for several symptoms, including short-term fatigue (32.34%), low back pain (31.60%), irritation (24.03%), headache (23.93%), and easily angered feelings (10.24%). Meanwhile, underweight individuals showed higher prevalence rates for abdominal pain (54.27%), rough skin (18.44%), mood swings (14.49%) and anxiety (14.12%).

Chi-square tests revealed significant differences in the prevalence of all symptoms across age groups. For BMI categories, significant differences were observed for most symptoms, except for certain physical symptoms (sleepiness, breast distending pain, dizziness) and emotional symptoms (depression and mood swings).

2. The results of GAM analysis.

**Fig. 5:**
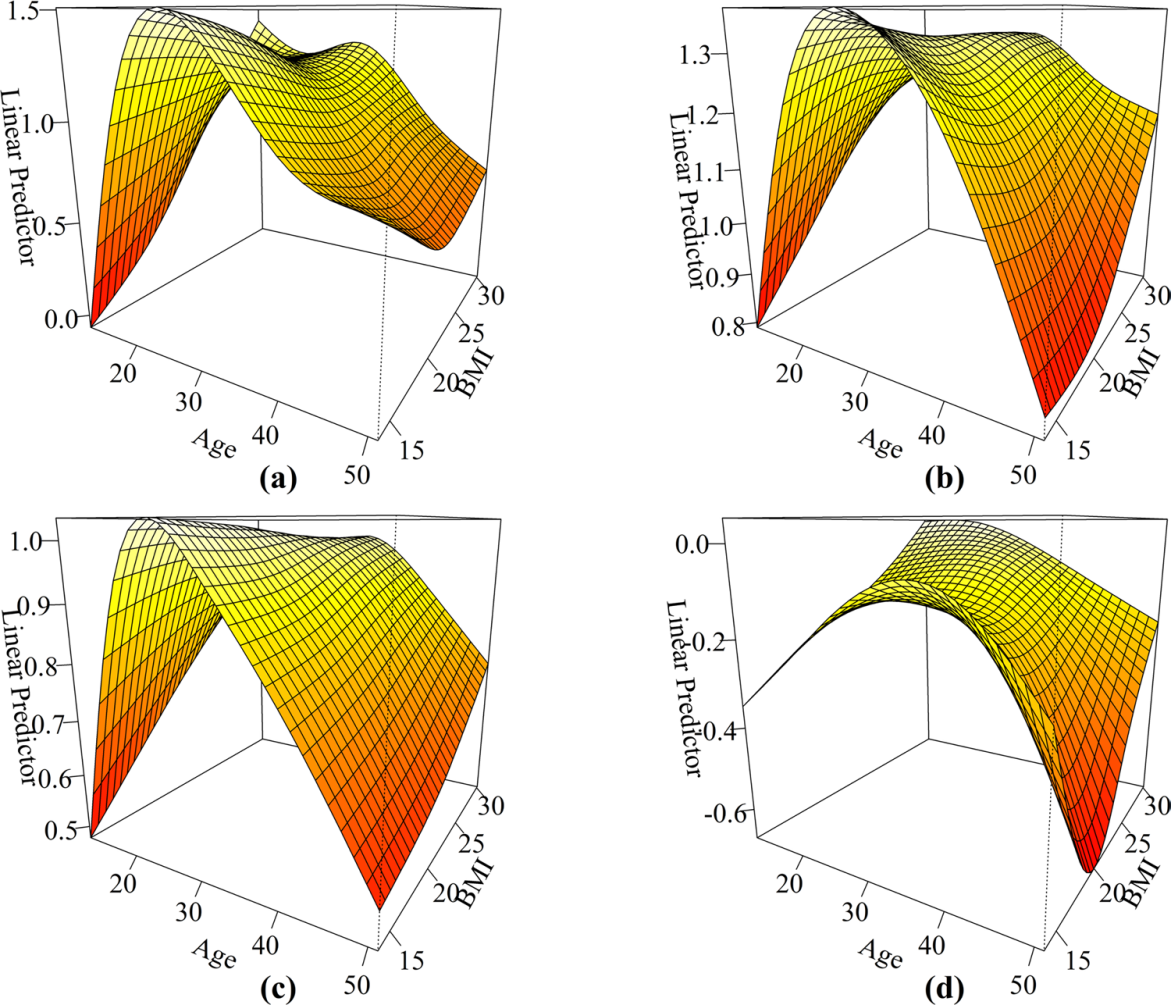
The complex nonlinear and interactive relationships between age, BMI, and menstrual pain (**a**), total number of symptoms (**b**), number of physical symptoms (**c**), and number of emotional symptoms (**d**).

Figure 5 presented the results GAM which was used to explore the complex nonlinear relationships between age, BMI, and the prevalence of menstrual pain (a), total menstrual symptoms (b), total physical menstrual symptoms (c), and total emotional menstrual symptoms (d). Age and BMI were treated as continuous variables, with the occurrence of menstrual pain as the primary binary response variable (where a score of 1 indicates no pain, and scores of 2, 3, and 4 represent mild, moderate, and severe pain, indicating the presence of menstrual pain).

All the smooth terms for the interaction between age and BMI were statistically significant (p < 0.0001). Visualizations revealed that age demonstrated a similar inverted U-shaped trend with all four response variables. The peaks for menstrual pain prevalence, number of total symptoms and physical symptoms were slightly left-skewed, while for number of emotional symptoms, the peak was centered at lower BMI values and shifted leftward as BMI increased. Additionally, as age increased, the relationship between the prevalence of menstrual pain, the number of total menstrual symptoms, physical symptoms, and emotional symptoms with BMI transitioned from a positive correlation to an inverted U-shape, and in most cases, an inverted U-shape was observed. At the same time, it is noteworthy that the overall curves for total symptoms and physical symptoms were relatively flat, showing a trend of being lower on the left and higher on the right.

**Fig. 6:**
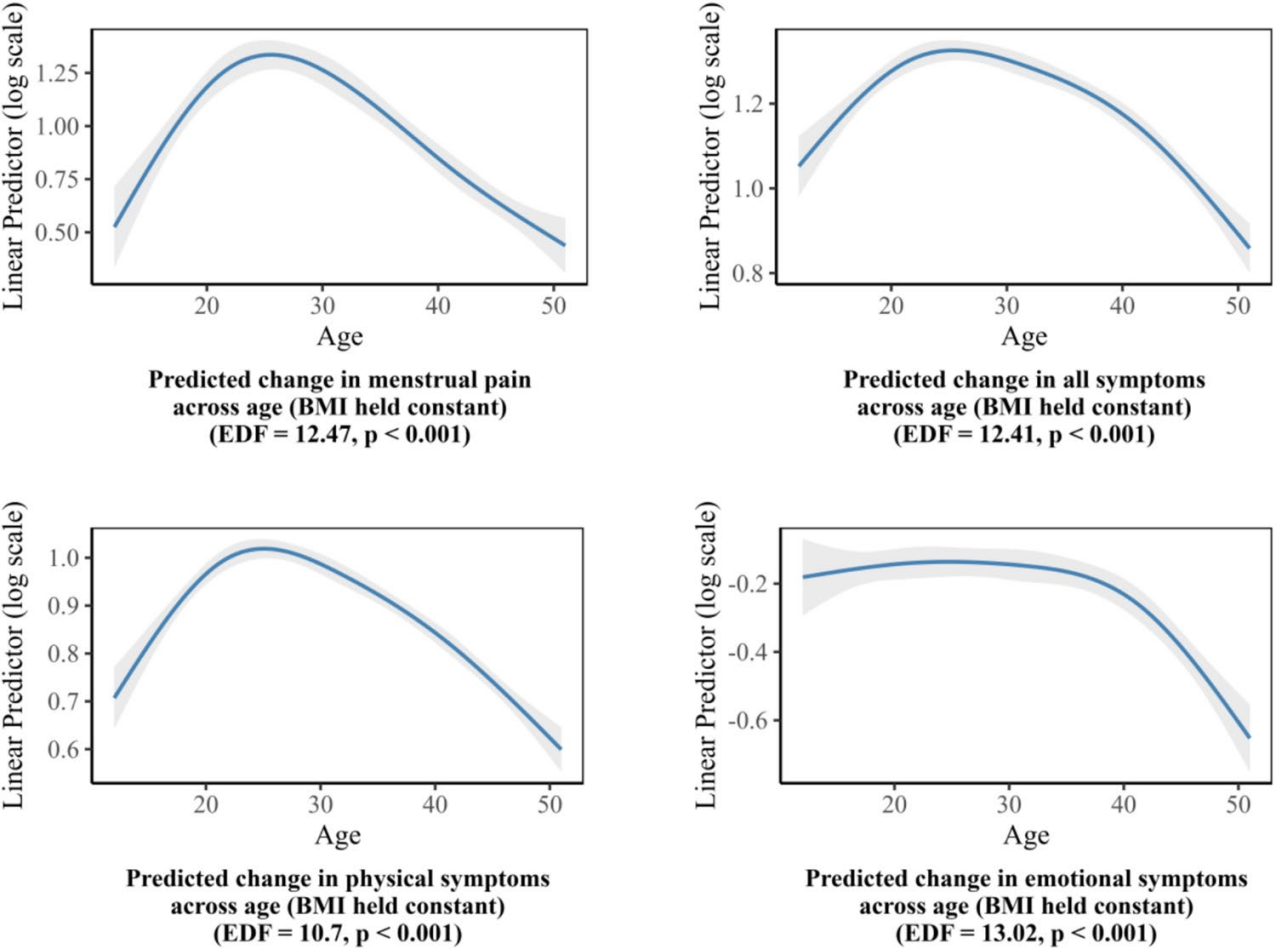
Estimated smoothing spline functions of age with 95% confidence bands from generalized additive models for menstrual-related outcomes, holding BMI constant.

Figure 6 illustrated the estimated smooth functions of age in relation to each outcome based on GAMs, with BMI held constant. Across all four outcomes—menstrual pain, total symptoms, physical symptoms, and emotional symptoms—age showed a statistically significant inverted U-shaped association (p < 0.001). Higher levels of menstrual pain, total symptoms, and physical symptoms were observed in the late twenties to early thirties, followed by a gradual decline at older ages. In contrast, emotional symptoms appeared to peak at a slightly older age and showed a more gradual change across the age range.

**Fig. 7:**
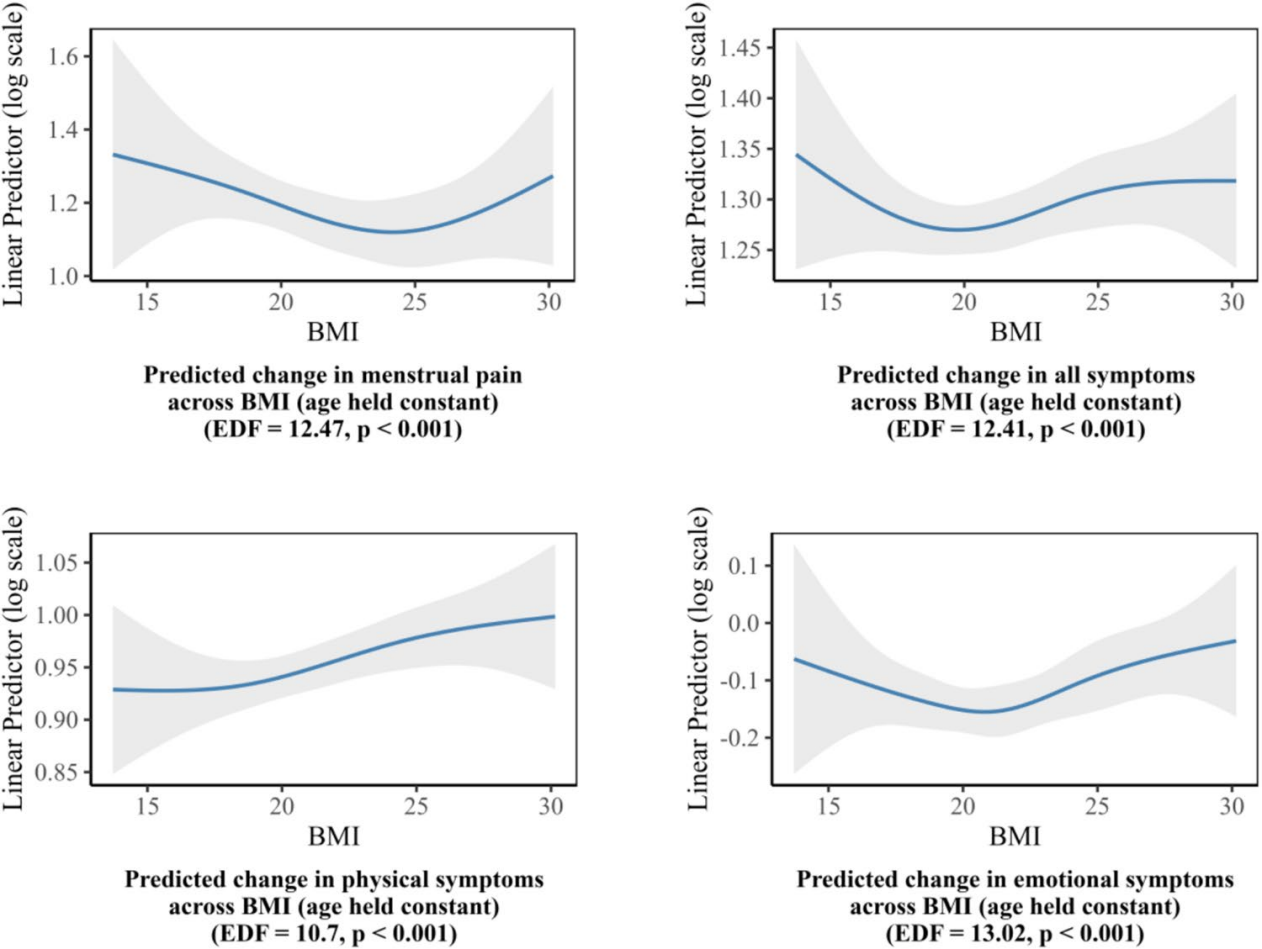
Estimated smoothing spline functions of BMI with 95% confidence bands from generalized additive models for menstrual-related outcomes, holding age constant.

Figure 7 presented the estimated smooth functions of BMI in relation to each outcome, with age held constant. All four relationships exhibited relatively shallow U-shaped patterns. The lowest levels of total symptoms and physical symptoms were observed at lower BMI values, followed by emotional symptoms. Menstrual pain showed its minimum at the highest end of the BMI distribution.

3. Sensitivity analysis

To address potential exclusion bias arising from the removal of participants using contraceptives or painkillers, a sensitivity analysis was conducted including these individuals. The results indicated that although BMI distributions did not differ significantly, the age distribution shifted, with a higher proportion of individuals aged 22–41 among those using contraceptives or painkillers. Additionally, participants using medication reported significantly greater menstrual pain and more menstrual-related symptoms, while the number of emotional symptoms remained comparable. These findings suggest that the excluded group had more severe symptoms and a different age structure, supporting their exclusion from the main analysis to maintain sample homogeneity and reduce potential confounding. Furthermore, the primary analyses were re-estimated using this broader sample, and the results did not differ significantly from the main analysis, indicating the robustness of the findings. These results are summarized in Supplementary Materials 4, 5, and 6, and a comparison of basic characteristics between groups is presented in Table 2.

**Table 2:** Summary of the basic characteristics between All participants (include people taking contraceptives or painkillers) and participants included in final analysis.

	All participants (include people taking pills or painkiller) (N = 42,461)	Participants included in final analysis (N = 32,556)	*p*-value
BMI group, n (row%)			
BMI: < 18.5	7447 (17.54%)	5641 (17.33%)	p = 0.23
BMI: 18.5–24.9	29,697 (69.94%)	22,746 (69.87%)	
BMI: 25–29.9	5231 (12.32%)	4083 (12.54%)	
BMI: > 30	86 (0.20%)	86 (0.26%)	
Age group, n (row%)			
12–21 years	8341 (19.64%)	6724 (20.65%)	p < 0.01
22–31 years	10,955 (25.80%)	8007 (24.59%)	
32–41 years	12,503 (29.45%)	9445 (29.01%)	
42–51 years	10,662 (25.11%)	8380 (25.74%)	
Menstrual pain, n (row%)			
No menstrual pain	8627 (20.32%)	8094 (24.86%)	p < 0.01
Mild menstrual pain	11,527 (27.14%)	9224 (28.33%)	
Moderate menstrual pain	12,035 (28.34%)	8181 (25.13%)	
Severe menstrual pain	4352 (10.25%)	4352 (13.37%)	
Unknown	3143 (7.40%)	2705 (8.31%)	
Number of all symptoms (mean; SD)	3.49 (2.66)	3.36 (2.66)	p < 0.01
Number of physical symptoms (mean)	2.57 (1.96)	2.43 (1.92)	p < 0.01
Number of emotional symptoms (mean)	0.82 (1.07)	0.82(1.07)	p = 0.23

## Discussion

This is the first large-scale study focusing on Japanese women to examine the relationship between age, BMI, and menstrual pain, along with other menstrual symptoms. With a sample size of 32,556 participants, the study demonstrates that that menstrual pain peaks at age 23 and follows an inverted U-shaped relationship with age, while a U-shaped relationship was observed with BMI.

The data collection was based on a mobile health application. In recent years, mobile health applications have been increasingly utilized in health tracking^28^ and interventions^29^, with many smartphone apps now available for managing menstrual pain and symptoms^28^. Mobile health app offers low costs and high accessibility, thereby expanding the reach of healthcare services^28,30^. Additionally, due to menstrual etiquette and stigma, women have often tended to conceal menstrual-related complaints^31,32^. Mobile health apps can help reduce stigma by providing an anonymous platform, thereby normalizing the symptoms experienced by individuals during menstruation^28,33^. This, in turn, enables participants to record their symptoms more accurately and authentically.

One of the key findings of this study is the high prevalence of menstrual pain among the Japanese women, with an overall rate of 66.83%, including 13.31% experiencing severe pain. These results align with previous studies, where menstrual pain prevalence ranged from 45 to 95%, depending on definitions and methods^13,22^. Similarly, the rate of severe pain is consistent with earlier findings, which reported 10–25% of reproductive-age women affected by very severe pain^7^. At the same time, the results of this study show that the prevalence of other menstrual symptoms ranged from 4.10% to 51.70%. This indicates that, in addition to menstrual pain, the prevalence of other menstrual-related symptoms is relatively high and warrants attention.

Apart from menstrual pain, this study also reported the distribution of other menstrual physical and emotional symptoms among Japanese women. The most commonly reported symptom among participants was abdominal pain. Gastrointestinal symptoms may be associated with the menstrual cycle. Previous studies suggest that the menstrual cycle and dysmenorrhea may be associated with functional gastrointestinal disorders^34^, such as irritable bowel syndrome^34^, functional dyspepsia^35^, and postprandial distress syndrome^35^. Although the mechanism of postprandial distress syndrome remains unclear, it may be linked to gastrointestinal motility changes triggered by hormonal fluctuations during menstruation^36^. Additionally, current evidence indicate that female pain sensitivity is influenced by cyclic changes in hormone levels^37^, particularly in women experiencing dysmenorrhea^7^. Additionally, menstrual pain may share overlapping pathophysiological mechanisms with chronic pain syndromes, such as central sensitization, peripheral sensibilization and abnormal stress response. Existing evidence from a systematic review and meta-analysis also indicates a strong association between dysmenorrhea and various chronic pain conditions^38^. These could also explain why pain-related symptoms, including abdominal pain (the most prevalent at 52.70%), lower back pain (third at 27.42%), headache (sixth at 23.77%), and breast distending pain (15th at 10.03%), were among the most frequently reported in this study.

In this study, sleepiness was the second most commonly reported symptom (27.53%), with similarly high proportions observed for short-term fatigue (26.18%) and long-term fatigue (16.89%). Although sleep status was not further assessed or clearly defined due to limitations in app-based data collection, these findings suggest a substantial burden of sleep-related complaints during menstruation. One possible explanation is that fluctuations in ovarian hormones across the menstrual cycle influence circadian rhythms—including changes in body temperature, melatonin secretion, and cortisol levels—which may reduce sleep quality and daytime alertness, contributing to the common experience of sleepiness and fatigue during menstruation^39,40^.

Depression was the most frequently reported emotional symptom during menstruation, with a prevalence of 26.26%. This may be attributed to the dysregulation of major stress-related central homeostatic systems, particularly the HPA axis^41^. Among women of reproductive age, estrogen and progesterone interact with the HPA axis and modulate its sensitivity and reactivity^42^. For instance, hormonal fluctuations across the menstrual cycle can alter cortisol responses, which in turn may either enhance or dampen stress reactivity, ultimately affecting mood regulation^43^. In parallel, studies have shown that women experiencing menstrual pain and other physical discomforts tend to report greater psychological distress, which is positively correlated with depressive and anxious symptoms^44,45^.

The results indicate that age is a contributing factor to menstrual pain and related symptoms. Although it is commonly believed that age is related to dysmenorrhea^8^, previous studies have yielded inconsistent conclusions regarding its influence on the prevalence of dysmenorrhea: A systematic review in 2014 consistently found a significant negative correlation between age and the risk of dysmenorrhea^46^. However, another systematic review and meta-analysis on factors associated with menstrual symptoms in 2023 identified being 20 years or older as a risk factor for primary dysmenorrhea^47^. This discrepancy may be due to previous studies categorizing participants into age groups for comparison, which can overlook the continuous nonlinear relationship between age and dysmenorrhea symptoms. In this study, we not only compared the prevalence of menstrual pain and the number of menstrual symptoms across age groups but also employed a Generalized Additive Model (GAM) to include age as a continuous variable. This approach provided a more nuanced perspective, revealing an inverted U-shaped relationship between age, menstrual pain prevalence, and the number of menstrual symptoms, thereby deepening our understanding of their interconnectedness. This relationship suggests that symptom severity tends to peak during the early to mid-reproductive years, typically between ages 20 and 30. Several physiological mechanisms may explain this pattern. During this period, the hypothalamic–pituitary–ovarian (HPO) axis is most active and hormonally dynamic, leading to greater fluctuations in estrogen and progesterone levels^48,49^. These hormonal shifts can enhance the synthesis of prostaglandins^50^, which are known to trigger stronger uterine contractions and contribute to menstrual pain^51,52^. Furthermore, both sex hormones and prostaglandins have been shown to modulate the production of inflammatory cytokines, potentially amplifying inflammatory responses associated with menstrual pain^53^. In addition, emerging evidence indicates that sex hormones are closely linked to individual differences in pain sensitivity, suggesting a broader role of hormonal regulation in the perception of menstrual pain^54^. In parallel, the inverted U-shaped association between age and menstrual pain observed in this study aligns with the age-related decline in Anti-Müllerian Hormone (AMH)^55,56^, a well-established marker of ovarian reserve that also peaks in early adulthood. Higher AMH levels during this period reflect an active follicular environment and increased ovarian function, which may contribute to greater cyclical hormonal variability and heightened endocrine reactivity^57^. Existing evidence has also reported a potential association between AMH and dysmenorrhea^58^. Additionally, as women age, a substantial proportion enter the childbearing phase, where parity has been shown to correlate negatively with menstrual pain and associated symptoms^46^.

Furthermore, this study demonstrated that BMI is a significant factor influencing the occurrence of menstrual pain and associated symptoms. Previous evidence have shown the relationships between BMI and the occurrence of dysmenorrhea, with underweight individuals possibly at increased risk of primary dysmenorrhea^11,47,59^. Additionally, several studies have suggested a potential link between overweight, obesity, and dysmenorrhea^60–62^. Body fat is an important extragonadal source of estrogen, and low body weight and reduced body fat can lead to hypothalamic dysfunction, affecting estrogen metabolism and the secretion of luteinizing hormone (LH) and follicle-stimulating hormone (FSH)^63^. At the same time, being overweight or obese may reduce the binding capacity of sex hormone-binding globulin, similarly disrupting hypothalamic function and causing hormonal imbalances^12,63,64^. Fluctuations in estrogen and progesterone can lead to excessive production of prostaglandins^65^, resulting in hypercontraction of the uterine muscles, which causes ischemia, hypoxia, and ultimately pain^63^. Additionally, evidence suggests that a higher BMI may be associated with systemic inflammatory responses^66^, and elevated inflammatory markers may have a potential link to the occurrence of dysmenorrhea^67,68^. Therefore, overweight and obesity may influence the development of dysmenorrhea by inducing inflammatory responses.

This study has the following limitations: (1) The study population consisted of users of a menstrual management app, who may have distinct characteristics compared to the general population, such as being younger or having greater health management needs^69,70^. This may limit the generalizability of the findings. However, our sampling scheme applies the sampling weight that corresponds to the general population. In this sense, the bias should be minimum. In addition, the study focused solely on the Japanese population, excluding individuals from other countries and ethnic backgrounds. Previous research has shown that the prevalence of menstrual pain may vary across regions and ethnicities^71^, which could lead to differences in the results. Future studies are needed to examine more diverse populations and settings to enhance the external validity of the findings. (2) The proportion of obese participants was very small compared to other BMI groups, which may have resulted in greater variability in the findings. This may be related to the extremely low obesity rate among Japanese women, which is only 1.6%^72^. (3) Menstrual symptoms were treated as binary variables, limiting the ability to explore the distribution of symptom severity and its relationship with age and BMI in depth. (4) As part of the Sofy app’s commitment to user privacy, information on several potential confounding factors—such as geographic region, marital status, physical activity, diet, hormone levels, underlying medical conditions, gynecological history (including surgery and reproductive history), sexual activity, comorbidities, work-related stress, and socioeconomic status—was not collected or stored. The absence of these variables may have limited the explanatory power of the reported models and constrained our ability to fully capture the complexity of menstrual symptom variability. Future studies should consider incorporating a broader range of biological, psychological, and social determinants to improve model accuracy and deepen the understanding of the underlying mechanisms of menstrual pain. (5) This study relied on app-based data collection and self-reported symptom records, which represents an inherent limitation. Subjective symptom reporting may vary due to individual differences in pain perception and thresholds, and is susceptible to biases such as social desirability, recall bias, and measurement error^73^. Moreover, app-based surveys may introduce reporting biases due to selective participation or misinterpretation of symptom categories^74,75^. Although online self-reporting remains a feasible and widely used method in large-scale epidemiological studies^76^, these potential biases should be considered when interpreting the findings. (6) Given the cross-sectional nature of this study, causal relationships between age, BMI, and menstrual symptoms cannot be established. While the observed associations offer important insights, longitudinal or experimental studies are warranted to clarify the temporal sequence and examine whether BMI and age-related factors may influence menstrual symptoms through mechanisms such as hormonal regulation, inflammation, or uterine contractility. (7) One limitation of this study is that participants who reported using contraceptives or painkillers were excluded from the main analysis. Although this approach aimed to reduce potential confounding from medication effects, it may have inadvertently excluded individuals with more severe menstrual symptoms, leading to a sample skewed toward milder cases. To address this concern, we conducted a sensitivity analysis including these participants. The results showed that while symptom severity was generally higher among medication users, the overall trends and associations observed in the primary analysis remained consistent, suggesting that the main findings are robust to this source of bias. (8) Due to limitations in the app-based questionnaire, participants were only asked whether they experienced sleepiness during menstruation, without further clarification on duration, timing (e.g., morning or daytime), or associated sleep behaviors (e.g., nighttime awakenings, sleep duration, or quality). This lack of granularity may obscure important distinctions between excessive daytime sleepiness, fatigue, and poor sleep quality. Consequently, this may introduce measurement imprecision and limit our understanding of the mechanisms underlying reported fatigue-related symptoms. Future studies should incorporate comprehensive assessments of sleep parameters, such as validated sleep questionnaires or actigraphy-based measures, to better capture the multidimensional nature of sleep disturbances and their impact on menstrual symptoms.

## Conclusion

In this study, a cross-sectional analysis was conducted on 32,556 Japanese female users of the Sofy app, aged 12 to 51. Among the participants, 66.83% reported experiencing menstrual pain, with 28.33% reporting mild pain, 25.13% moderate pain, and 13.31% severe pain. Additionally, other menstrual symptoms were highly prevalent, with participants reporting an average of 3.36 symptoms (SD: 2.66). We further investigated the nonlinear relationships between age, BMI, and both menstrual pain and related symptoms. Notably, the prevalence of menstrual pain and the number of symptoms demonstrated an inverted U-shaped trend with age and a U-shaped trend with BMI. In previous studies, comparisons were often made between different age groups and BMI categories, overlooking the potential nonlinear relationship between age, BMI, and menstrual pain and symptoms when treated as continuous variables. This study offers a unique perspective on the relationship between age, BMI, menstrual pain, and the number of menstrual symptoms, which can further enhance understanding and provide a scientific foundation for identifying high-risk groups, thereby informing future targeted interventions and treatment strategies.

## Supplementary Information

Supplementary Information.

## Data Availability

The datasets used and analyzed during the current study are not publicly available due to proprietary restrictions imposed by Sofy. However, the data can be made available by the corresponding author upon reasonable request and with permission from Sofy Company.
