# Supplementary material for: Prevalence of menstrual pain and symptoms and their association with age and BMI among Japanese female app users
Source: Sci Rep. 2025 May 28;15:18743. doi: 10.1038/s41598-025-02459-0 (PMC12119975; doi:10.1038/s41598-025-02459-0)

**Prevalence of Menstrual Pain and Menstrual Symptoms in Japan: Based on 32,556 Responses from menstrual track app**

**Shuxian Liu^1^**

**Daisuke Yoneoka^2^**

**Hitomi Suzuki^3^**

**Kiriko Sasayama^4^**

**Eiko Saito^4^**

**Yuna Naraoka^5, 6, 7^**

**Momo Hosokawa^7^**

**Erika Ota^1*^**

**1. Global Health Nursing, Graduate School of Nursing Science, St. Luke's International University, Tokyo, Japan.**

**2. Center for Surveillance, Immunization, and Epidemiologic Research, National Institute of**

**Infectious Diseases, Tokyo,Japan**

**3. International Healthcare and Midwifery, Graduate School of Nursing, Japanese Red Cross College of Nursing**

**4. Sustainable Society Design Center, Graduate School of Frontier Sciences, The University of Tokyo, Chiba, Japan**

**5. Intractable Disease Research Center, Graduate School of Medicine, Juntendo University, 2-1-1 Hongo, Bunkyo-ku, Tokyo 113-8421, Japan**

**6. Japanese Center for Research on Woman in Sport, Juntendo University, 2-1-1 Hongo, Bunkyo-ku, Tokyo 113-8421, Japan**

**7. General Incorporated Association Luvtelli, 3-2-14, Nihonbashi, Chou-ku, Tokyo 103-0027, Japan**

***** **Corresponding author. Global Health Nursing, Graduate School of Nursing Science, St. Luke's International University, Tokyo, Japan.**

**E-mail address: [ota@slcn.ac.jp](mailto:ota@slcn.ac.jp)(Erika Ota)**

**Supplementary material 1. Model Fit Comparison Using AIC and BIC Across Four Symptom Outcomes**

| outcome | Model | AIC | BIC |
| --- | --- | --- | --- |
| Menstrual Pain | Linear | 34758.90 | 34783.81 |
|  | Polynomial | 34542.64 | 34584.16 |
|  | Spline | 34491.10 | 34565.83 |
|  | GAM | 34490.30 | 34575.35 |
| All Symptoms | Linear | 121345.78 | 121370.18 |
|  | Polynomial | 120724.58 | 120765.24 |
|  | Spline | 120699.41 | 120772.60 |
|  | GAM | 120648.09 | 120761.10 |
| Physical Symptoms | Linear | 124889.63 | 124914.73 |
|  | Polynomial | 124431.72 | 124473.55 |
|  | Spline | 124378.82 | 124454.10 |
|  | GAM | 124328.10 | 124420.49 |
| Emotional Symptoms | Linear | 66026.81 | 66051.31 |
|  | Polynomial | 65914.22 | 65955.04 |
|  | Spline | 65911.68 | 65985.17 |
|  | GAM | 65913.57 | 65992.34 |

**Supplementary material 1: Prevalence and 95% CIs of various symptoms across different age and BMI groups.**

| Group | Symptom | Prevalence | CI_Lower | CI_Upper |
| --- | --- | --- | --- | --- |
| overweight | short term fatigue | 0.33 | 0.31 | 0.34 |
| overweight | abdominal pain | 0.52 | 0.50 | 0.54 |
| overweight | rough skin | 0.14 | 0.12 | 0.15 |
| overweight | sleepiness | 0.28 | 0.26 | 0.29 |
| overweight | breast distending pain | 0.10 | 0.09 | 0.11 |
| overweight | headache | 0.24 | 0.22 | 0.25 |
| overweight | low back pain | 0.32 | 0.30 | 0.33 |
| overweight | chills | 0.11 | 0.10 | 0.12 |
| overweight | edema | 0.16 | 0.15 | 0.17 |
| overweight | increased appetite | 0.11 | 0.10 | 0.12 |
| overweight | long term fatigue | 0.21 | 0.19 | 0.22 |
| overweight | nausea | 0.04 | 0.03 | 0.04 |
| overweight | dizziness | 0.04 | 0.03 | 0.05 |
| overweight | irritation | 0.24 | 0.23 | 0.26 |
| overweight | easily angered | 0.10 | 0.09 | 0.11 |
| overweight | depression | 0.28 | 0.26 | 0.30 |
| overweight | anxiety | 0.11 | 0.10 | 0.12 |
| overweight | mood swings | 0.14 | 0.13 | 0.15 |
| underweight | short term fatigue | 0.28 | 0.27 | 0.30 |
| underweight | abdominal pain | 0.54 | 0.53 | 0.56 |
| underweight | rough skin | 0.18 | 0.17 | 0.20 |
| underweight | sleepiness | 0.29 | 0.28 | 0.30 |
| underweight | breast distending pain | 0.11 | 0.10 | 0.12 |
| underweight | headache | 0.22 | 0.21 | 0.23 |
| underweight | low back pain | 0.28 | 0.26 | 0.29 |
| underweight | chills | 0.13 | 0.12 | 0.14 |
| underweight | edema | 0.13 | 0.12 | 0.14 |
| underweight | increased appetite | 0.11 | 0.10 | 0.12 |
| underweight | long term fatigue | 0.17 | 0.16 | 0.18 |
| underweight | nausea | 0.05 | 0.05 | 0.06 |
| underweight | dizziness | 0.05 | 0.04 | 0.06 |
| underweight | irritation | 0.21 | 0.19 | 0.22 |
| underweight | easily angered | 0.09 | 0.08 | 0.10 |
| underweight | depression | 0.27 | 0.26 | 0.28 |
| underweight | anxiety | 0.14 | 0.13 | 0.15 |
| underweight | mood swings | 0.14 | 0.13 | 0.16 |
| normal weight | short term fatigue | 0.29 | 0.28 | 0.30 |
| normal weight | abdominal pain | 0.52 | 0.51 | 0.53 |
| normal weight | rough skin | 0.16 | 0.16 | 0.17 |
| normal weight | sleepiness | 0.29 | 0.28 | 0.30 |
| normal weight | breast distending pain | 0.11 | 0.10 | 0.11 |
| Group | Symptom | Prevalence | CI_Lower | CI_Upper |
| normal weight | headache | 0.22 | 0.22 | 0.23 |
| normal weight | low back pain | 0.28 | 0.27 | 0.29 |
| normal weight | chills | 0.12 | 0.11 | 0.12 |
| normal weight | edema | 0.16 | 0.16 | 0.17 |
| normal weight | increased appetite | 0.12 | 0.12 | 0.13 |
| normal weight | long term fatigue | 0.18 | 0.17 | 0.18 |
| normal weight | nausea | 0.04 | 0.04 | 0.05 |
| normal weight | dizziness | 0.04 | 0.04 | 0.04 |
| normal weight | irritation | 0.20 | 0.20 | 0.21 |
| normal weight | easily angered | 0.08 | 0.08 | 0.09 |
| normal weight | depression | 0.26 | 0.26 | 0.27 |
| normal weight | anxiety | 0.13 | 0.12 | 0.13 |
| normal weight | mood swings | 0.14 | 0.13 | 0.14 |
| obesity | short term fatigue | 0.27 | 0.15 | 0.39 |
| obesity | abdominal pain | 0.42 | 0.29 | 0.56 |
| obesity | rough skin | 0.04 | -0.01 | 0.09 |
| obesity | sleepiness | 0.27 | 0.15 | 0.39 |
| obesity | breast distending pain | 0.13 | 0.04 | 0.23 |
| obesity | headache | 0.12 | 0.03 | 0.20 |
| obesity | low back pain | 0.19 | 0.09 | 0.30 |
| obesity | chills | 0.08 | 0.00 | 0.15 |
| obesity | edema | 0.13 | 0.04 | 0.23 |
| obesity | increased appetite | 0.06 | -0.01 | 0.12 |
| obesity | long term fatigue | 0.25 | 0.13 | 0.37 |
| obesity | nausea | 0.06 | -0.01 | 0.12 |
| obesity | dizziness | 0.06 | -0.01 | 0.12 |
| obesity | irritation | 0.10 | 0.02 | 0.18 |
| obesity | easily angered | 0.04 | -0.01 | 0.09 |
| obesity | depression | 0.38 | 0.25 | 0.52 |
| obesity | anxiety | 0.10 | 0.02 | 0.18 |
| obesity | mood swings | 0.12 | 0.03 | 0.20 |
| Age 12-21 | short term fatigue | 0.26 | 0.24 | 0.27 |
| Age 12-21 | abdominal pain | 0.56 | 0.54 | 0.57 |
| Age 12-21 | rough skin | 0.19 | 0.18 | 0.20 |
| Age 12-21 | sleepiness | 0.28 | 0.27 | 0.29 |
| Age 12-21 | breast distending pain | 0.12 | 0.11 | 0.13 |
| Age 12-21 | headache | 0.19 | 0.18 | 0.20 |
| Age 12-21 | low back pain | 0.28 | 0.27 | 0.29 |
| Age 12-21 | chills | 0.12 | 0.11 | 0.13 |
| Age 12-21 | edema | 0.13 | 0.12 | 0.14 |
| Age 12-21 | increased appetite | 0.15 | 0.15 | 0.16 |
| Age 12-21 | long term fatigue | 0.12 | 0.11 | 0.13 |
| Age 12-21 | nausea | 0.06 | 0.05 | 0.06 |
| Group | Symptom | Prevalence | CI_Lower | CI_Upper |
| Age 12-21 | dizziness | 0.04 | 0.04 | 0.05 |
| Age 12-21 | irritation | 0.22 | 0.21 | 0.23 |
| Age 12-21 | easily angered | 0.09 | 0.08 | 0.10 |
| Age 12-21 | depression | 0.26 | 0.25 | 0.28 |
| Age 12-21 | anxiety | 0.14 | 0.13 | 0.15 |
| Age 12-21 | mood swings | 0.15 | 0.15 | 0.16 |
| Age 22-31 | short term fatigue | 0.30 | 0.29 | 0.31 |
| Age 22-31 | abdominal pain | 0.59 | 0.58 | 0.60 |
| Age 22-31 | rough skin | 0.22 | 0.21 | 0.23 |
| Age 22-31 | sleepiness | 0.33 | 0.32 | 0.34 |
| Age 22-31 | breast distending pain | 0.14 | 0.13 | 0.15 |
| Age 22-31 | headache | 0.21 | 0.20 | 0.22 |
| Age 22-31 | low back pain | 0.31 | 0.30 | 0.32 |
| Age 22-31 | chills | 0.14 | 0.13 | 0.15 |
| Age 22-31 | edema | 0.18 | 0.17 | 0.19 |
| Age 22-31 | increased appetite | 0.13 | 0.13 | 0.14 |
| Age 22-31 | long term fatigue | 0.20 | 0.19 | 0.21 |
| Age 22-31 | nausea | 0.06 | 0.05 | 0.06 |
| Age 22-31 | dizziness | 0.05 | 0.04 | 0.05 |
| Age 22-31 | irritation | 0.20 | 0.19 | 0.21 |
| Age 22-31 | easily angered | 0.08 | 0.07 | 0.08 |
| Age 22-31 | depression | 0.31 | 0.29 | 0.32 |
| Age 22-31 | anxiety | 0.15 | 0.14 | 0.16 |
| Age 22-31 | mood swings | 0.16 | 0.15 | 0.17 |
| Age 32-41 | short term fatigue | 0.32 | 0.30 | 0.33 |
| Age 32-41 | abdominal pain | 0.51 | 0.50 | 0.53 |
| Age 32-41 | rough skin | 0.15 | 0.14 | 0.16 |
| Age 32-41 | sleepiness | 0.30 | 0.29 | 0.32 |
| Age 32-41 | breast distending pain | 0.10 | 0.09 | 0.11 |
| Age 32-41 | headache | 0.24 | 0.23 | 0.25 |
| Age 32-41 | low back pain | 0.30 | 0.29 | 0.31 |
| Age 32-41 | chills | 0.12 | 0.11 | 0.13 |
| Age 32-41 | edema | 0.17 | 0.16 | 0.18 |
| Age 32-41 | increased appetite | 0.11 | 0.10 | 0.11 |
| Age 32-41 | long term fatigue | 0.21 | 0.20 | 0.22 |
| Age 32-41 | nausea | 0.04 | 0.03 | 0.04 |
| Age 32-41 | dizziness | 0.04 | 0.04 | 0.05 |
| Age 32-41 | irritation | 0.22 | 0.21 | 0.23 |
| Age 32-41 | easily angered | 0.10 | 0.09 | 0.11 |
| Age 32-41 | depression | 0.27 | 0.26 | 0.28 |
| Age 32-41 | anxiety | 0.12 | 0.11 | 0.13 |
| Age 32-41 | mood swings | 0.13 | 0.12 | 0.14 |
| Age 42-51 | short term fatigue | 0.27 | 0.15 | 0.39 |
| Group | Symptom | Prevalence | CI_Lower | CI_Upper |
| Age 42-51 | abdominal pain | 0.42 | 0.29 | 0.56 |
| Age 42-51 | rough skin | 0.04 | -0.01 | 0.09 |
| Age 42-51 | sleepiness | 0.27 | 0.15 | 0.39 |
| Age 42-51 | breast distending pain | 0.13 | 0.04 | 0.23 |
| Age 42-51 | headache | 0.12 | 0.03 | 0.20 |
| Age 42-51 | low back pain | 0.19 | 0.09 | 0.30 |
| Age 42-51 | chills | 0.08 | 0.00 | 0.15 |
| Age 42-51 | edema | 0.13 | 0.04 | 0.23 |
| Age 42-51 | increased appetite | 0.06 | -0.01 | 0.12 |
| Age 42-51 | long term fatigue | 0.25 | 0.13 | 0.37 |
| Age 42-51 | nausea | 0.06 | -0.01 | 0.12 |
| Age 42-51 | dizziness | 0.06 | -0.01 | 0.12 |
| Age 42-51 | irritation | 0.10 | 0.02 | 0.18 |
| Age 42-51 | easily angered | 0.04 | -0.01 | 0.09 |
| Age 42-51 | depression | 0.38 | 0.25 | 0.52 |
| Age 42-51 | anxiety | 0.10 | 0.02 | 0.18 |
| Age 42-51 | mood swings | 0.12 | 0.03 | 0.20 |

**Supplementary material 3. *p* values of various symptoms across different age and BMI groups.**

| Symptom | p value for age group | p value for BMI group |
| --- | --- | --- |
| Short Term Fatigue | < 0.001 | < 0.001 |
| Abdominal Pain | < 0.001 | < 0.001 |
| Rough Skin | < 0.001 | < 0.001 |
| Sleepiness | < 0.001 | 0.829 |
| Breast Distending Pain | < 0.001 | 0.156 |
| Headache | < 0.001 | 0.003 |
| Low Back Pain | < 0.001 | < 0.001 |
| Chills | < 0.001 | < 0.001 |
| Edema | < 0.001 | < 0.001 |
| Increased Appetite | < 0.001 | 0.005 |
| Long Term Fatigue | < 0.001 | < 0.001 |
| Nausea | < 0.001 | 0.007 |
| Dizziness | 0.012 | 0.062 |
| Irritation | < 0.001 | < 0.001 |
| Easily Angered | < 0.001 | 0.002 |
| Depression | < 0.001 | 0.077 |
| Anxiety | < 0.001 | < 0.001 |
| Mood Swings | < 0.001 | 0.33 |

**Supplementary material 3. Bar chart of the prevalence of menstrual physical and emotional symptoms, including users of contraceptives and painkillers, with 95% confidence intervals.**


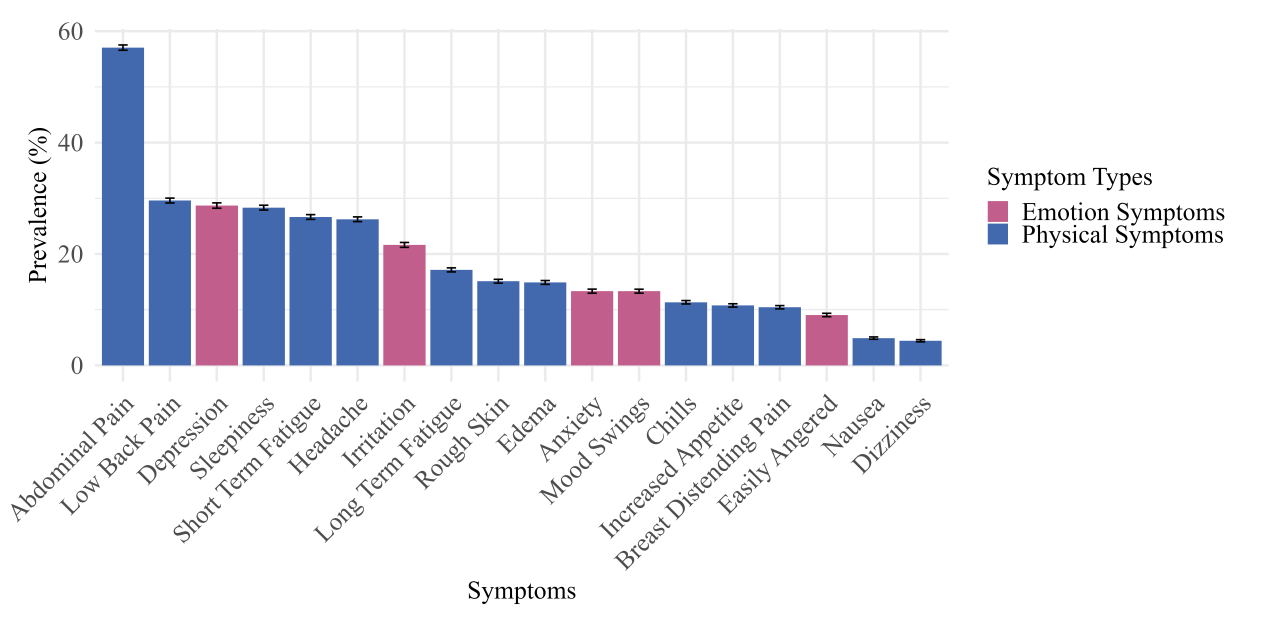


**Supplementary material 4. The distribution of menstrual pain across different age groups (a) and BMI categories (b), and the number of menstrual symptoms across different age groups (c) and BMI categories (d) with 95% CI, including users of contraceptives and painkillers, with 95% confidence intervals.**


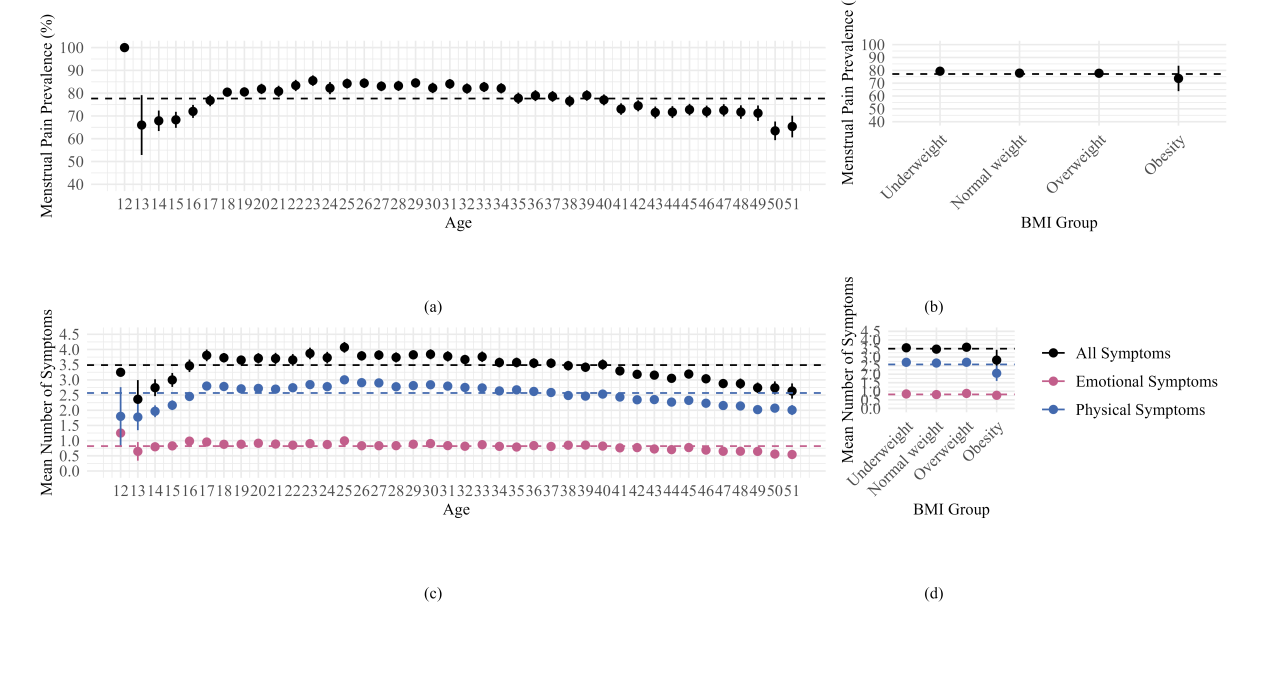


**Supplementary material 5. Symptom Prevalence Distribution Radar Chart: (a) Radar chart showing symptom prevalence distribution across age groups; (b) Radar chart showing symptom prevalence distribution across BMI groups, including users of contraceptives and painkillers, with 95% confidence intervals.**


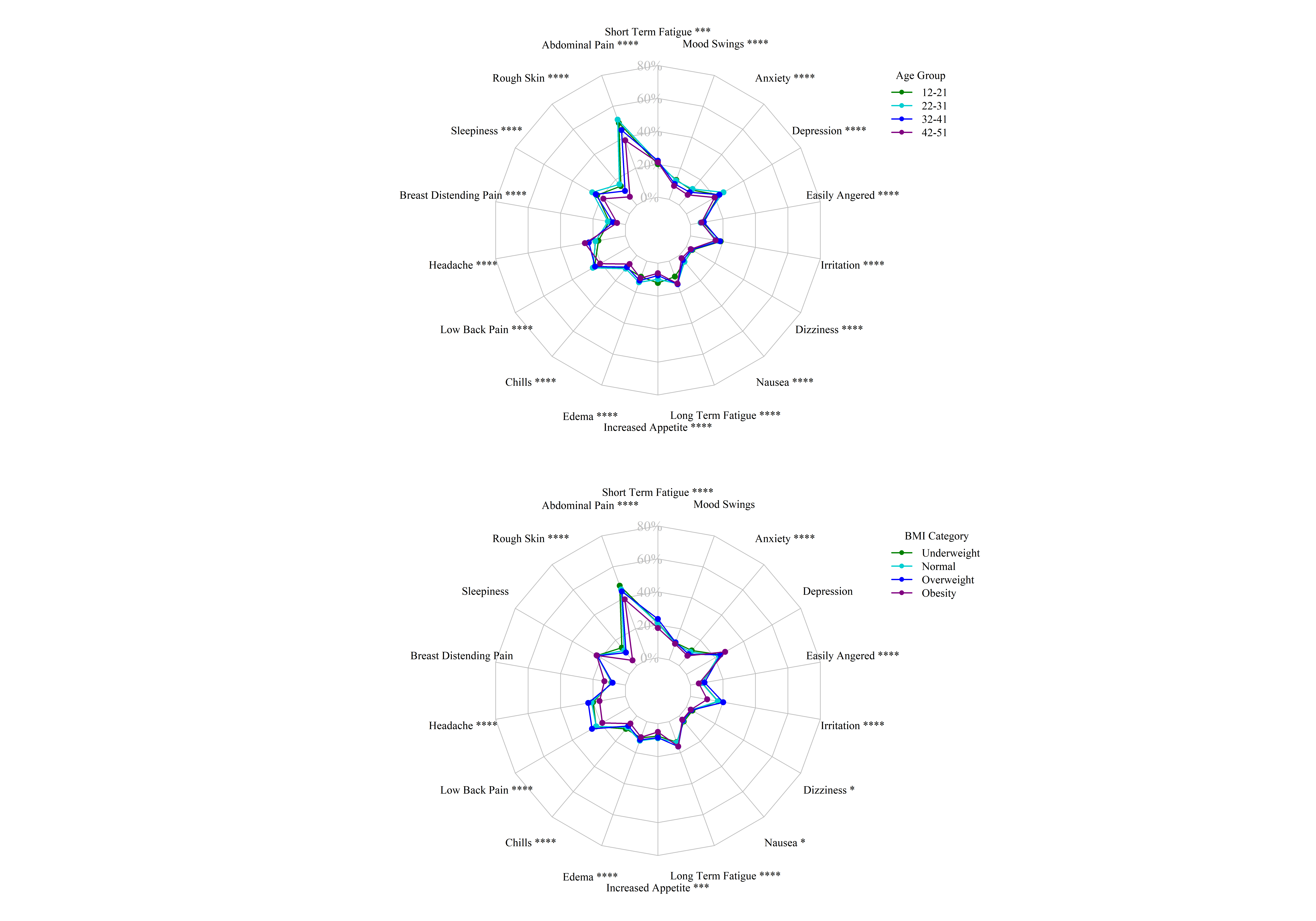


**Supplementary material 6. the complex nonlinear and interactive relationships between age, BMI, and menstrual pain(a), total number of symptoms(b), number of physical symptoms(c), and number of emotional symptoms(d), including users of contraceptives and painkillers, with 95% confidence intervals.**


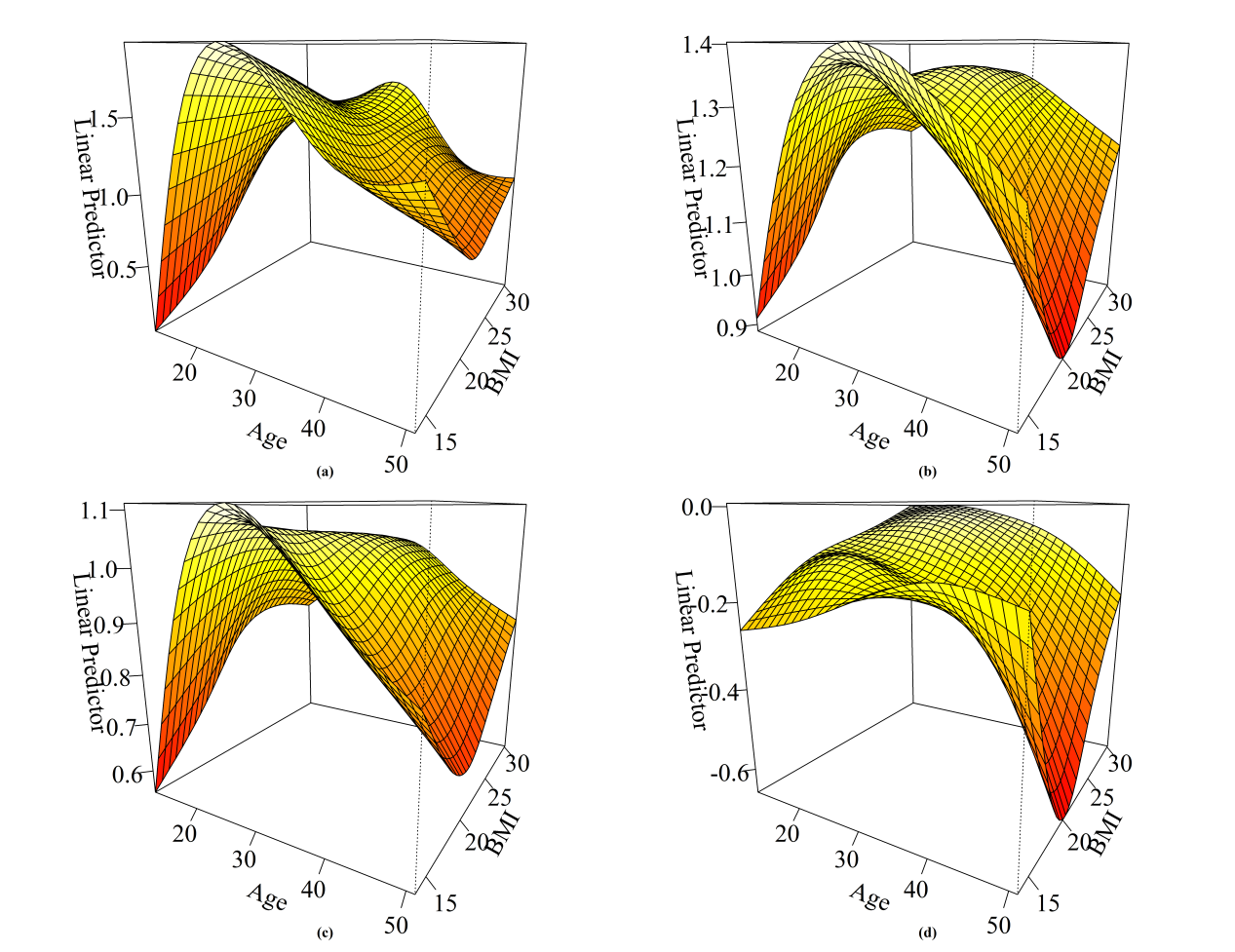

Supplement: Supplementary file 1 — Supplementary Information. [file 41598_2025_2459_MOESM1_ESM.docx]
